# Long-Term Oncological Outcomes Following Anastomotic Leakage After Colorectal Cancer Surgery: A Retrospective Monocenter Trial

**DOI:** 10.7759/cureus.92431

**Published:** 2025-09-16

**Authors:** Mario Kaufmann, Julia Hardt, Christopher Bozinov, Lothar Pilz, Steffen Seyfried, Christoph Reissfelder, Georgi Vassilev, Georgi Kalev

**Affiliations:** 1 Department of Surgery, Universitätsmedizin Mannheim, Medical Faculty Mannheim, Heidelberg University, Mannheim, DEU; 2 Department of Biostatistics and Epidemiology, Universitätsmedizin Mannheim, Medical Faculty Mannheim, Heidelberg University, Mannheim, DEU

**Keywords:** anastomotic leakage, colorectal cancer, colorectal cancer surgery, long-term outcome, overall survival (os)

## Abstract

Background and objectives: Colorectal cancer (CRC) ranks among the most common cancers worldwide. One of the most severe postoperative complications is an anastomotic leakage (AL). The aim of this study was to investigate the impact of AL on the long-term oncologic outcome.

Methods: All patients who underwent curative surgery for CRC at the Department of Surgery at the University Hospital in Mannheim between January 2011 and June 2016 were retrospectively analyzed. The primary endpoint was the overall survival (OS).

Results: Between January 2011 and June 2016, a total of 504 patients were included in the study. The median follow-up was 42 months. We found an AL incidence of 11.1% (n = 56) with the following risk factors: age ≤ 65 (p = 0.03) and location of the tumor in the rectum, especially mid-rectum (p = 0.012). Patients with AL were less likely to have adjuvant chemotherapy (p = 0.002). AL had no significant impact on the OS (p = 0.09). Patients with AL had a significantly higher 90-day mortality (p = 0.002), whereas there was no significant impact on local or distant recurrence.

Conclusion: In our cohort, age ≤ 65 and a cancer location in the mid-rectum were risk factors for AL. However, our results did not demonstrate a significant influence of AL on the oncological long-term outcome.

## Introduction

Colorectal cancer (CRC) is one of the leading causes of cancer death worldwide. In the United States, it is the third most common cause of cancer-related death in both men and women. CRC is the second most common malignant tumor in women and the third most common in men in German-speaking countries. The average age of onset is 70-75 years. However, there has been an age shift in recent years: increasingly younger patients are developing CRC; one-fifth of patients are in their early 50s or even younger when they develop CRC [1].

Although the treatment of CRC-especially rectal cancer-is increasingly multimodal, surgical oncological resection remains the mainstay of curative therapy. Probably the most important and still the most feared complication after CRC surgery is anastomotic leakage (AL). AL is responsible for one-third of postoperative mortality after surgery for rectal cancer [2]. Accordingly, 30-day mortality has been shown to be significantly higher for patients with AL (8.8% vs. 2.5%) [3].

Studies have identified several risk factors for the occurrence of AL. It was shown that the localization of the anastomosis plays an important role: especially, low rectal anastomoses have a high risk of AL. Moreover, male gender and preoperative radiotherapy may increase the risk of AL [4]. Further independent predictors of AL are obesity, emergency surgery, absence of a diverting ileostomy, American Society of Anesthesiologists (ASA) score ≥ III, preoperative tumor complications, extensive additional (multivisceral) resection, and preoperative hypoalbuminemia indicating malnutrition [5,6].

However, despite standardized double-stapled anastomosis technique and perioperative measures to reduce patient risk factors (nutritional advice, patient blood management, prehabilitation programs, combined bowel preparation, etc.), AL remains a relevant complication that leads to further potentially severe secondary morbidity. In addition, AL could be associated with a poorer oncological long-term outcome for the affected patients [7]. The aim of this study was to determine whether AL is associated with overall survival (OS), recurrence rates, and postoperative mortality using a large monocentric cohort from a CRC center certified by the German Cancer Society and a center of excellence for coloproctology of the German Society for General and Visceral Surgery.

## Materials and methods

Study design, location, and setting

This retrospective cohort study was designed in accordance with the Declaration of Helsinki. After approval (2020-844R) from the institutional ethics committee, the Ethics Committee II of the University of Heidelberg, Medical Faculty Mannheim, the study was conducted at the Department of Surgery at the University Hospital in Mannheim, Germany.

Patient recruitment

All patients who were at least 18 years old and underwent curative surgery for CRC between January 2011 and June 2016 were included in the analysis.

Inclusion and exclusion criteria

In order not to distort the survival statistics, patients who had never received curative treatment and were never tumor-free were excluded. On the other hand, patients in Union for International Cancer Control (UICC) stage IV were also included if they only had oligometastatic disease, i.e., if they could be treated curatively. Furthermore, all patients who did not undergo primary restoration of intestinal continuity by means of intestinal anastomosis-such as those who underwent Hartmann’s procedure or abdominoperineal resection-were excluded.

Definition of AL

AL was defined and graded according to the proposal by the International Study Group of Rectal Cancer [8]. Even if this classification was initially proposed for rectal cancer, it seems reasonable to adopt it also for AL occurring after resection for colon cancer. This pragmatic approach can also be found in comparable studies [9]. However, it must be taken into account that AL occurring after colon resection is usually not asymptomatic (grade A) and in most cases cannot be managed using non-surgical interventions (grade B, e.g., endoscopic vacuum therapy and CT-guided percutaneous drainage), but is very likely to require repeat surgery (grade C). Thus, after colon resection, a significantly higher proportion of grade C AL in the total number of AL cases is to be expected. In cases of clinical and/or laboratory suspicion of AL, a CT scan was initially performed in accordance with our internal hospital standards. In cases of rectal resection, a sigmoidoscopy was also performed if necessary.

Surgical procedures

All patients scheduled for elective oncological colorectal resection with primary anastomosis were eligible for inclusion in the study, regardless of the choice of surgical approach (laparoscopic, open, or robotic). The study cohort contains only very few robot-assisted procedures, as robotic surgery was only established at the Department of Surgery at the University Hospital in Mannheim in 2016. All resections included complete mesocolic excision (CME), partial mesocolic excision (PME), or total mesorectal excision (TME), depending on the tumor location in the colon and rectum. After colon resection, the bowel was reconstructed either intracorporeally as a side-to-side stapler anastomosis or extracorporeally as an end-to-end circular hand suture anastomosis. Reconstruction after rectal resection was routinely performed by creating a double-stapled side-to-end anastomosis using a 28 or 29 circular stapler. A protective ileostomy was generally performed in accordance with our hospital standard. The standard reconstruction technique after intersphincteric resection for ultralow rectal cancer patients was end-to-end handsewn coloanal anastomosis. All patients were monitored in accordance with the German guideline for CRC using regular CT scans, colonoscopies, clinical examinations, and carcinoembryonic antigen (CEA) measurements.

Study endpoints

The primary endpoint of the study was OS. The survival statistics were calculated as follows: OS refers to the patient's survival since the date of surgery. The end of the calculation period was the date on which the patient died, regardless of the underlying cause. Disease-free survival (DFS) is defined as a period of freedom from disease during which the patient survives without any symptoms of the tumor. This period begins with the date of surgery if it leads to tumor freedom and ends with the first date a disease recurrence (local or distant) is diagnosed or the last date of follow-up if no recurrence occurs. The secondary endpoints of the study were recurrence (including both local and distant recurrences) and 30- and 90-day mortality.

Statistical analysis

The patient characteristics were given in absolute numbers and percentages with the corresponding confidence intervals (CIs). The 95% CI was calculated using the modified Wald test.

Survival statistics were calculated using the Kaplan-Meier method. The log-rank test was used to compare the survival times of two groups. The calculation of the CIs of the survival statistics as a “function” of an AL was carried out using two different methods, described by Brookmeyer and Crowley and by Reid [10,11]. The K-sample test was used to test the homogeneity of the samples. The local and distant recurrence rates, 30- and 90-day mortality, and corresponding subgroup analyses were carried out using the chi-squared distribution test. For the plausibility checks of data, MS Excel (Microsoft Office LTSC Professional Plus 2021; Microsoft, Redmond, WA, US) was used. For the analysis of ordinal data and survival data, we used SAS University Edition Software, Version 9.4 (SAS Inc., Cary, NC, US). For the calculation of the CIs of proportions and ordinal data, GraphPad Prism was used, version 9.3 (GraphPad Software, Dotmatics, Boston, MA, US). Categorical variables are expressed as absolute frequencies and proportions (%); CIs are given using the Wald method and compared between two groups using the χ² tests. The odds ratio (OR), 95% CI, and p-value are calculated according to Altman using the 2 x 2 table of events/non-events in each of the groups. All significance tests were two-tailed with type I error set at α = 0.05.

## Results

Between January 2011 and June 2016, a total of 504 patients underwent curative surgery for CRC with preservation of bowel continuity at the Department of Surgery at the University Hospital in Mannheim, Germany, and could therefore be included in the present study. The median age was 66 years, with an age range from 27 to 97 years. The study cohort consisted of 60.5% men, whereas women were in the minority with the remaining 39.5%. The most common tumor location was the mid-rectum with 23%. Overall, almost half of the tumors (45.6%) were in the rectum, whereby tumors of the rectosigmoid junction were not included here but were evaluated as colon carcinomas. Most diagnoses were made in UICC stage III with 42.1%, followed by UICC stage I with 27.4% and II with 26.6%. The majority of operations (54.8%) were performed conventionally laparoscopically, 43.3% of patients underwent open surgery, and only 0.6% underwent robot-assisted surgery. Further patient, tumor, and procedure characteristics can be found in Table 1.

**Table 1:** Patient, tumor, and surgical procedure characteristics of the study cohort (n = 504) CI: confidence interval; UICC: Union for International Cancer Control

	Abs. number (n = 504)	Percentage (%)	95% CI
Age (years)			
≤65	237	47.0	0.427-0.514
>65	267	53.0	0.486-0.573
Sex			
Male	305	60.5	0.562-0.647
Female	199	39.5	0.353-0.438
Location of cancer			
No information	1	0.2	<0.001-0.012
Cecum	55	10.9	0.085-0.140
Ascending colon	74	14.7	0.119-0.181
Transverse colon	28	5.6	0.038-0.079
Descending colon	12	2.4	0.013-0.042
Sigmoid	86	17.1	0.140-0.206
Rectosigmoid	1	0.2	<0.001-0.012
Upper rectum	43	8.5	0.064-0.113
Mid-rectum	116	23.0	0.196-0.269
Lower rectum	71	14.1	0.113-0.174
UICC stage			
No information	3	0.6	0.001-0.018
I	138	27.4	0.237-0.314
II	134	26.6	0.229-0.306
III	212	42.1	0.378-0.464
IV	17	3.4	0.021-0.054
Molecular grading			
Not applicable	29	5.8	0.040-0.082
G1	21	4.2	0.027-0.063
G2	385	76.4	0.725-0.799
G3	66	13.1	0.104-0.163
G4	3	0.6	0.001-0.018
Surgical approach			
No information	7	1.4	0.006-0.029
Open	218	43.3	0.390-0.476
Laparoscopic	276	54.8	0.504-0.591
Robotic	3	0.6	0.001-0.018
Type of surgery			
No information	2	0.4	0.001-0.015
Right hemicolectomy	129	25.6	0.220-0.296
Transverse colectomy	7	1.4	0.006-0.029
Left hemicolectomy	26	5.2	0.035-0.075
Sigmoid resection	45	8.9	0.067-0.118
Subtotal colectomy	15	3.0	0.018-0.049
Proctocolectomy	15	3.0	0.018-0.049
Anterior resection	68	13.5	0.108-0.168
Low anterior resection	185	36.7	0.326-0.410
Ultralow/intersphincteric resection	12	2.4	0.013-0.042
Radiotherapy			
Neoadjuvant	139	27.58	0.239-0.316
Adjuvant	7	1.39	0.006-0.029
None	358	71.03	0.669-0.748

AL and associated factors

Of the 504 patients in the study cohort, 56 patients suffered AL (11.1%). Grade B AL was the most common AL type (4.8%). Grade C AL was similarly common (4.2%). Grade A AL was the least frequently diagnosed (1.8%). To determine the risk factors associated with AL in this study cohort, subgroup analyses were carried out (Table 2). The comparison of the cohorts with and without AL showed that most patients with AL (60.7%) were significantly younger, i.e., under 65 years of age. In contrast, 54.6% of the cohort of patients without AL were over 65 years old (p = 0.030). Furthermore, the occurrence of AL was found to be significantly dependent on the tumor location. AL occurred most frequently after resection of tumors of the mid-rectum (41.1%). In general, AL occurred more frequently after resection of rectal carcinoma than after colon carcinoma resection (69.6%, p = 0.012). Even though the proportion of patients with UICC stages III and IV or with ASA class IV was higher in the cohort with AL, UICC stage and ASA class were not significantly associated with the development of AL.

**Table 2:** Subgroup analyses of factors associated with the occurrence of anastomotic leakage ASA: American Society of Anesthesiologists; CI: confidence interval; UICC: Union for International Cancer Control *χ² tests

	Leakage (-) (n = 445)		Leakage (+) (n = 56)		p
	n (%)	95% CI	n (%)	95% CI	
Age					
≤65	202 (45.39)	0.408-0.500	34 (60.71)	0.476-0.724	0.030*
>65	243 (54.61)	0.500-0.592	22 (39.29)	0.276-0.524	
UICC stage					
No information	1 (0.22)	<0.001-0.014	0 (0.00)	-	0.824*
I	123 (27.64)	0.237-0.320	14 (25.00)	0.154-0.378	
II	121 (27.19)	0.233-0.315	13 (23.21)	0.680-0.888	
III	186 (41.80)	0.373-0.464	26 (46.43)	0.340-0.593	
IV	14 (3.15)	0.018-0.053	3 (5.36)	0.013-0.152	
ASA class					
0	4 (0.90)	0.003-0.024	0 (0.00)	-	0.458*
1	107 (24.04)	0.203-0.282	12 (26.79)	0.126-0.340	
2	222 (49.89)	0.453-0.545	30 (53.57)	0.407-0.660	
3	106 (23.82)	0.201-0.280	9 (16.07)	0.085-0.280	
4	6 (1.35)	0.006-0.029	2 (3.57)	0.003-0.128	
Location of cancer					
Cecum	53 (11.91)	0.092-0.153	1 (1.79)	<0.001-0.103	0.012*
Ascending colon	69 (15.51)	0.124-0.192	5 (8.93)	0.035-0.197	
Transverse colon	27 (6.07)	0.042-0.087	1 (1.79)	<0.001-0.103	
Descending colon	12 (2.70)	0.015-0.047	0 (0.00)	-	
Sigmoid	78 (17.53)	0.143-0.214	8 (14.29)	0.072-0.260	
Rectosigmoid	16 (3.60)	0.022-0.058	2 (3.57)	0.003-0.128	
Upper rectum	36 (8.09)	0.059-0.110	7 (12.50)	0.059-0.239	
Mid-rectum	93 (20.90)	0.174-0.249	23 (41.07)	0.292-0.541	
Lower rectum	61 (13.71)	0.108-0.172	9 (16.07)	0.085-0.280	

Primary outcomes

**Figure 1:**
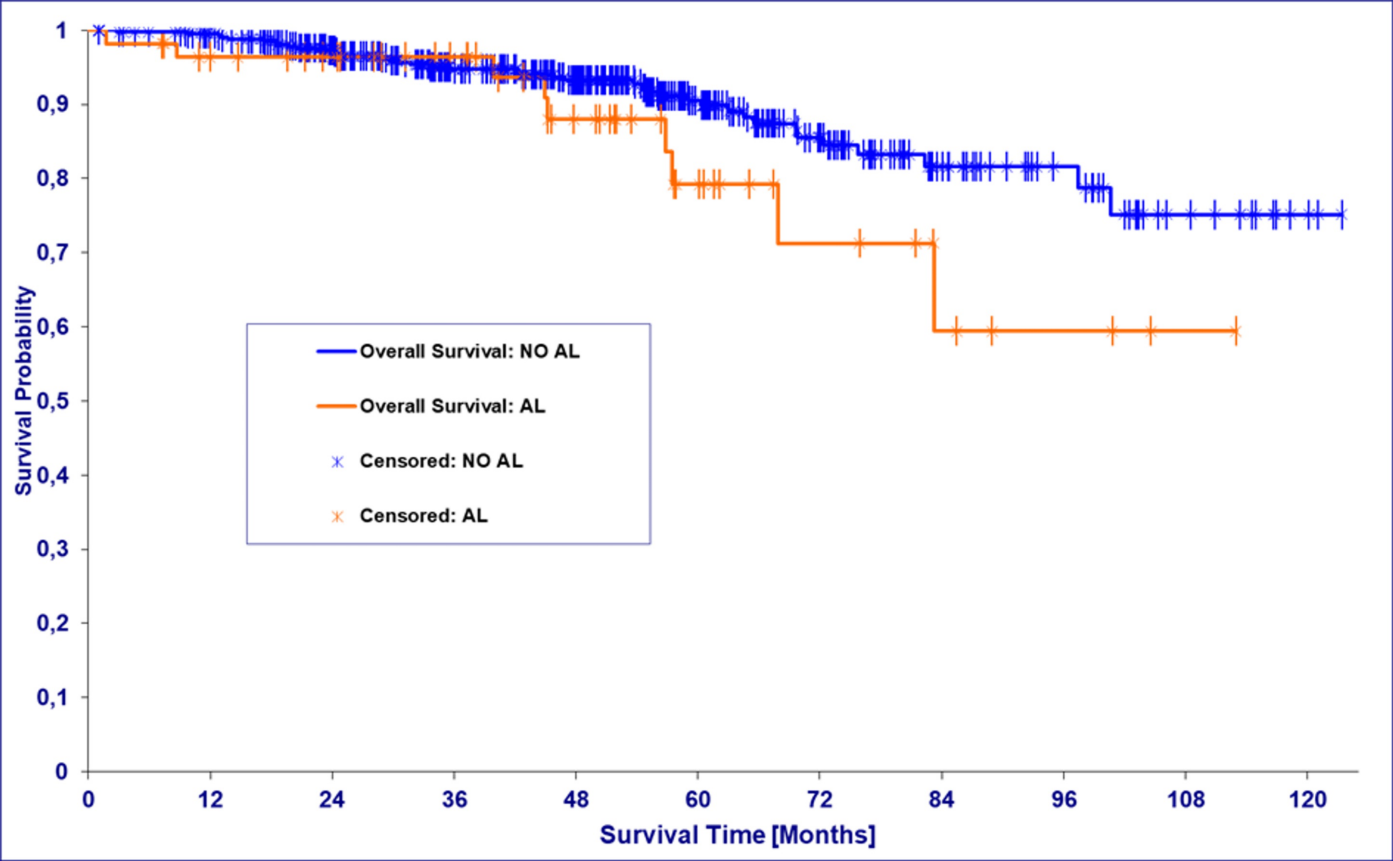
Kaplan-Meier estimate: overall survival with or without anastomotic leakage (AL)

Initially, the survival probability of patients with AL is only slightly worse. After about 3.5 years, however, there is a clear divergence of the two survival curves to the disadvantage of the cohort with AL (Figure 1). Patients with an AL had a median OS of 60.3 months (95% CI 53.7-72.4), and patients without an AL had a median OS of 45 months (95% CI 39.9-83.2) (Table 3).

**Table 3:** Correlation of the primary and secondary endpoints with the occurrence of anastomotic leakage OS: overall survival; CI: confidence interval *Log-rank test **χ² tests

	Leakage (-) (n = 445)	Leakage (+) (n = 56)	p
OS			
Median	60.3	45.2	0.090**
95% CI	53.7-72.4	39.9-83.2	
Local recurrence			
n (%)	25 (5.6)	1 (1.8)	0.223*
95% CI	0.038-0.082	<0.001-0.103	
Distant recurrence			
n (%)	74 (16.6)	11 (19.6)	0.571*
95% CI	0.135-0.204	0.112-0.320	
30-day mortality			
n (%)	1 (0.2)	1 (1.8)	0.081*
95% CI	<0.001-0.014	<0.001-0.103	
90-day mortality			
n (%)	1 (0.2)	2 (3.8)	0.002*
95% CI	<0.001-0.014	0.112-0.320	

Secondary outcomes

The median follow-up regarding the oncological endpoints was 42 months (range 1-114). Sufficient data were available from 501 patients for the evaluation of recurrences in relation to the occurrence of AL. Local recurrences occurred in 5.4% of the patients (26/501). Distant recurrences were even more frequent, affecting 17.5% of the patients (85/501). Of the 56 patients with AL, one patient (1.8%) developed a local recurrence and 11 patients (19.6%) a distant recurrence. In contrast, 25 of the 445 patients without AL (5.6%) had a local recurrence, and 74 patients (16.6%) had a distant recurrence. There was no statistically significant difference regarding recurrence rates between the AL and non-AL cohorts (local recurrence: p = 0.223; distant recurrence: p = 0.571). Furthermore, we assessed both 30- and 90-day postoperative mortality. One patient died in each of the two cohorts with and without AL within 30 days after surgery (AL: 1/56 (1.8%) vs. non-AL: 1/445 (0.2%); p = 0.081). Two patients with AL died within 90 days of surgery (3.8%), whereas there was no further death in the non-AL cohort between postoperative days 30 and 90. Thus, significantly more patients died within 90 days if AL had occurred (p = 0.002).

## Discussion

This single-center retrospective cohort study investigated the factors associated with AL and the influence of AL on the oncological outcome of patients with CRC after curative surgery at a certified center of excellence for CRC surgery. A total of 504 patients were included in the study. The AL incidence of 11.1% (n = 56) is comparable to similar studies [12,13]. Hasegawa et al. described in a retrospective cohort (n = 395) an incidence of 12.7% for rectal cancer patients with UICC classes I-III [13]. The analyses of the Netherlands Cancer Register (n = 65,299 colon cancer; n = 22,855 rectal cancer) by Arron et al. showed an AL incidence of 4.8% (n = 3,136) and 7.9% (n = 1,814) for colon and rectal cancers, respectively [12].

Our study revealed that patients with AL were significantly younger (<65 years), and the localization of the tumor also played a decisive role. Patients with a tumor in the mid-rectum had a significantly higher AL rate than patients with colon cancer. The results regarding age are controversial in the literature: While Stormark et al. (n = 22,985 colon cancer) showed a higher risk of AL in older patients (>79 years), Arron et al. described a higher risk in younger patients (<70 years) [9,12]. A smaller analysis by Hasegawa et al. (n = 395) showed no differences regarding the impact of patient age on the risk of AL. An extensive resection of locally advanced tumors can potentially be associated with an increased risk for AL. The evidence regarding the localization of the tumor as a risk factor for AL is also heterogeneous, whereby the different classifications should be noted here in particular. When considering colon carcinoma alone, tumors distal to the right flexure or transverse resections and subtotal colectomies appear to be particularly at risk of AL [9,12].

It was shown in this study, comparable to the literature, that patients with AL were significantly less likely to receive planned adjuvant chemotherapy [12]. Patients with a non-complicated postoperative course could reach a faster return to intended oncologic treatment (RIOT). Postoperative complications in oncologic liver and gastric surgery are risk factors for an inability to RIOT, which in turn is correlated with a shortness of OS [14,15].

The median OS was 45.2 months in the AL group compared to 60.3 months in the non-AL group, presumably reflecting the significantly higher proportion of patients over 65 years of age in the non-AL group. There was no statistically significant impact of the occurrence of an AL on OS. However, there was a divergence of the Kaplan-Meier curves after 3.5 years in favor of the patients who did not develop AL. Significant effects for this parameter were shown in meta-analyses [16,17]. Regarding five-year relative survival, large registry analyses showed a significant disadvantage for patients with AL in the entire cohort [9]. It is to be expected that we would also have seen this effect with a larger number of cases. The subgroup analyses according to the UICC classification and tumor location showed that colon cancer patients in UICC stage III and rectal cancer patients in UICC stages III and IV had a lower survival rate after AL [9,12].

Our study could not identify AL as a significant risk factor for local and distant tumor recurrence. This is in part in contrast with meta-analyses that showed a significant increase in local recurrence but not in distant recurrence after AL [16,17]. Generally, no local tumor cell contamination is to be expected after R0 resection in the case of AL. In patients who suffer AL after resection of a locally advanced CRC potentially preceded by neoadjuvant radiotherapy or radiochemotherapy (rectal cancer), indicated adjuvant chemotherapy is often delayed or not administered at all. This may be a possible explanation for the correlation between AL and local recurrence in patients treated according to the traditional treatment paradigms. Future studies will show whether the application of complete chemotherapy prior to surgery in the sense of total neoadjuvant therapy (TNT) would limit the impact of AL on the recurrence rate in rectal cancer patients.

Another common theory is that AL leads to localized inflammation and is, therefore, potentially more likely to result in recurrence, as the local immune competence that fights the inflammation is at least partially reduced [18]. Regarding 30- and 90-day mortality rates, the 90-day mortality rate was significantly higher after the occurrence of AL. Although a significant difference was found (p = 0.002), only a few patients died within the first 90 days after surgery: two patients (3.8%) in the AL group vs. one patient (0.2%) in the non-AL group.

Future studies must take a closer look at the pathophysiological effects of AL, preventive measures, early detection, and treatment methods. The identification of patients at risk is the decisive factor in preventing AL through closer monitoring. If AL and its secondary complications can be successfully prevented, more patients will be able to continue their intended oncological therapy, which could be associated with an improvement in prognosis.

Limitations

The retrospective design and its relatively small number of cases, which results in insufficient statistical power, limit the study. While the T4 stage and the presence of lymph node metastases, as known risk factors for local recurrence, were assessed by recording the UICC stage, data on other risk factors such as R0 status and, in patients with rectal cancer, a positive circumferential margin or extramural venous invasion were not collected [16,19-21]. It was, therefore, not possible to compare the groups with and without AL regarding these risk factors, which represents a potential source of bias. Similar to some other authors, we analyzed the OS and the rate of local and distant recurrence as measures of long-term outcome, but we did not evaluate disease-free and cancer-specific survival since the data required to calculate these parameters were not available [22-24]. Furthermore, this study examined data collected between January 2011 and June 2016, so the impact of more recently proposed treatment strategies such as immunotherapy or TNT in patients with rectal cancer was not evaluated. In this cohort study, AL did not significantly affect OS or recurrence, but further prospective studies with multivariable adjustment are needed.

## Conclusions

The occurrence of AL continues to be a serious complication in the postoperative course after colorectal surgery. In our study cohort, young patients and patients with tumors located in the mid-rectum were particularly frequently affected. AL had a significant impact on 90-day mortality. However, the occurrence of AL did not significantly influence the OS.
